# Impact of formalin fixation on mismatch repair protein evaluation by immunohistochemistry

**DOI:** 10.1007/s00428-023-03661-z

**Published:** 2023-09-29

**Authors:** Federica Grillo, Murad Ali, Michele Paudice, Simona Pigozzi, Giorgia Anselmi, Stefano Scabini, Stefania Sciallero, Nataniele Piol, Luca Mastracci

**Affiliations:** 1 Pathology Unit, IRCCS Ospedale Policlinico San Martino, Genoa, Italy; 2 Pathology Unit, Department of Surgical Sciences and Integrated Diagnostics (DISC), University of Genoa and IRCCS Ospedale Policlinico San Martino, Genoa, Italy; 3 Molecular Diagnostic Unit, IRCCS Ospedale Policlinico San Martino, Genoa, Italy; 4 Oncological Surgical Unit, IRCCS Ospedale Policlinico San Martino, Genoa, Italy; 5 Medical Oncology Unit 1, IRCCS Ospedale Policlinico San Martino, 16132 Genoa, Italy

**Keywords:** Microsatellite instability, Mismatch repair proteins, Colorectal cancer, Pre-analytic variables, Formalin fixation

## Abstract

Mismatch repair/microsatellite instability (MMR/MSI) status in colorectal cancer (CRC) has become fundamental as a diagnostic, prognostic, and predictive factor. MMR immunohistochemistry (IHC) is considered a simple and reliable approach; however, its effectiveness depends on pre-analytic factors. Aim of this study was to investigate the impact of different fixation times/protocols on MMR protein IHC quality. Left over tissue from surgically resected CRC samples (cold ischemia time < 30 min) where fixed as follows: standard formalin fixation (24–48 h); hypo-fixation (<20 h); hyper-fixation (>90 h); cold (4°C) fixation (24–48 h); standard fixation for small sample size (0.5×0.5 cm). Samples for each group were collected from 30 resected CRC and the following parameters were evaluated on 600 immunohistochemical stains: intensity of expression; patchiness of staining; presence of central artefact. Forty-six immunoreactions were inadequate (score 0 intensity), the majority regarding MLH1 or PMS2 in the hypo-fixation group (47.8%), followed by the hyper-fixation group (28.1%); cold formalin fixation showed the least inadequate cases. Patchiness and central artefact were more frequent in hypo-fixation and standard fixation group compared to the others. MLH1 (closely followed by PMS2) performed worse with regard to immunostaining intensity (*p*=0.0002) in the standard and in the hypo-fixation group (*p*< 0.00001). Using a small sample size improved patchiness/central artefacts. This is the first study specifically created to evaluate the impact of fixation on MMR protein IHC, showing that both formalin hypo- and hyper-fixation can cause problems; 24-h formalin fixation as well as cold (4°C) formalin fixation are recommended for successful IHC MMR evaluation.

## Introduction

The highly conserved DNA mismatch repair (MMR) complex plays a crucial role in preserving genomic stability by identifying and correcting DNA mismatches, insertions and deletions that can occur during DNA replication. Deficient MisMatch Repair (dMMR) tumors are characterized by a high spontaneous mutation rate caused by defects in one of the 4 MMR genes (MLH1, PMS2, MSH2, MSH6) and EPCAM. Lynch syndrome (LS) is due to germline mutations of the MMR genes [1] while somatic mutations and, more frequently MLH1 promotor hypermethylation, lead to epigenetic silencing in sporadic, non-familial, colorectal cancer (CRC) and endometrial cancer.

Defects in the MMR complex can be identified using three different testing strategies. The less expensive, faster, and more accessible approach is the evaluation of MMR proteins (MLH1, PMS2, MSH2, and MSH6) by immunohistochemistry (IHC) on formalin-fixed and paraffin-embedded (FFPE) tumor samples [2, 3], while a PCR-based assay or Next Generation Sequencing approach, which identify Microsatellite Instability (MSI), are generally reserved for problematic cases [4].

From a clinical perspective, MMR screening/MSI testing has many advantages in CRC: (i) universal screening in CRC and endometrial cancer is being implemented for the identification of LS families [5–7]; (ii) stage II/III CRCs should be evaluated for dMMR/MSI status as they show better prognosis [8] and knowledge of MMR/MSI status influences the choice of adjuvant chemotherapy [9]; (iii) immune checkpoint inhibitor therapy has been approved for metastatic dMMR/MSI CRCs or recurrences [10].

Routine MMR IHC is being performed on all newly resected CRC specimens in many institutions and it provides a simple and reliable approach. Indeed, the majority of pathology labs are well equipped for IHC; nevertheless, the effectiveness of IHC depends on how the tissue is handled in terms of pre-analytic factors [11–13]. The impact of pre-analytic factors has been extensively studied in some cancer types, less so in others [14–16].

MMR/MSI testing is becoming ever more important in the clinical and therapeutic management of CRC and a reliable test result is fundamental. A relatively recent study by Cohen et al. has, however, shown that primary resistance to immune checkpoint inhibitor therapy may be due to errors in MMR/MSI evaluation and this was seen in 10% of patients with metastatic CRC who had been recruited for treatment with a false-positive dMMR or MSI-PCR result determined by local laboratories [17]. The reasons behind these errors are variable, including pre-analytical factors (which are by far the most frequent), assay-related factors, and interpretation problems giving rise to possibly discrepant results [18].

Cold ischemia time and formalin fixation time/process have a major impact on IHC in the pre-analytic stage [19, 20] and this is especially true in large resection specimens (less so in small biopsies which are usually quickly immersed in formalin) [21, 22]. Fixation is an essential step in tissue processing, and both under and over-fixation of surgical samples can result in poor nucleic acid quality, and inconclusive DNA/RNA analysis and can impact IHC by lowering the intensity and extent of immunostaining [13, 23, 24]. Other reasons for pre-analytic variability include hypoxia related-factors (e.g., in pre-treated colorectal liver metastases or neoadjuvant treatment for example in locally advanced rectal cancer [25]) as well as long-term archival preservation of FFPE blocks or unstained sections [26, 27]. A recent study shows how cold (4°C) formalin fixation ensures high-quality DNA, out-performing standard room temperature fixation and its use in antigen preservation for IHC could also be effective [28, 29].

The present study aims to investigate the impact of formalin hypo- and hyperfixation on IHC for MMR proteins by using different fixation protocols on left-over tissue from surgically resected CRC specimens with known cold ischemia times. A further aim was to investigate whether cold (4°C) formalin fixation could improve IHC quality.

## Materials and methods

### Sample collection

Sample accrual was performed at the Unit of Anatomic Pathology, University of Genova and IRCCS San Martino Polyclinic Hospital, Genova Italy from surgically resected CRC specimens with the following inclusion criteria:

1. Only non-fixed, fresh, CRC resected cases;
2. Large dimensions of the CRC (only left-over tissue was collected after sampling for diagnosis);
3. Samples sent to the Anatomic Pathology laboratory with known cold ischemia times of less than 30 min;
4. CRC which had not undergone prior neoadjuvant therapy.

### Study protocol

From each selected neoplasm, 5 samples were taken and fixed in 10% neutral buffered formalin as follow:

- Group A: Standard: formalin fixation at room temperature between 24 and 48 h; dimensions 2×1 cm.
- Group B: Hypo-fixation in formalin at room temperature < 20 h; 2×1 cm dimensions.
- Group C: Hyper-fixation in formalin at room temperature > 90 h; dimensions 2×1 cm
- Group D: Cold (4°C) formalin fixation between 24 and 48 h; dimensions 2×1 cm.
- Group E: Standard small: formalin fixation at room temperature between 24 and 48 h; dimensions 0.5×0.5 cm.

The normal samples were taken as best of care dictates, ensuring that samples included areas of deepest invasion, as well as areas with non-neoplastic mucosa. Immunostaining was performed on the selected tissue block, avoiding samples with large areas of necrosis or mucin. The small samples were taken from the invasive edges and the central (often more necrotic) areas were avoided; where possible non-neoplastic tissue was included in the small sample.

All groups were composed of 30 samples each. Room temperature formalin fixed samples were routinely processed. Cold formalin fixed samples were immediately immersed in 4°C pre-chilled formalin and kept at 4°C for fixation time. Samples were dehydrated in 4°C pre-chilled 95% ethanol for 4 h and then sent to processing using the standard processing program on Leica ASP6025S processor (Leica Microsystems, Wetzlar, Germany) starting from the second ethanol step [29].

### Immunohistochemistry protocols

Immunohistochemistry was performed on all samples for four MMR proteins: MLH1, PMS2, MSH2 and MSH6. Immunoreactions were performed using the automated BenchMark ULTRA immunostainer (Ventana Medical Systems, Tucson, Arizona, USA). See Table 1 for antibody clones and protocols.

**Table 1:** Immunohistochemistry specifications for mismatch repair proteins (MLH1, MSH2, PMS2, MSH6) with specifications for clone, pre-treatment, incubation and development on the Ventana BenchMark ULTRA immunostainer (Ventana Medical Systems, Tucson, Arizona, USA)

	MLH1	PMS2	MSH2	MSH6
Manufacturer	VENTANA anti-MLH1	VENTANA anti-MSH2	VENTANA anti-MSH6	VENTANA anti-PMS2
Clone	M1	A16-4	G219-1129	SP93
Heat pretreatment	64 min	92 min	36 min	36 min
Antibody incubation	80 min at room temperature	32 min room temperature	60 min at room temperature	20 min at 37°C
Development system	ULTRAVIEW DAB IHC	OPTIVIEW DAB IHC	ULTRAVIEW DAB IHC	ULTRAVIEW DAB IHC

### Evaluation of IHC slides

All immunostained slides were evaluated by expert gastrointestinal pathologists with ten-year experience in Universal MMR screening (FG, LM). Positive controls were available (on-slide controls [30] and as internal controls - normal colonic glands or stroma/lymphocytes).

Complete loss or preservation of nuclear expression of MMR proteins permitted categorization as MMR deficient (dMMR) or proficient (pMMR) and was performed for each CRC on the standard sample (A) [31, 32]. In short, dMMR was defined as complete loss of nuclear expression of tumor nuclei with maintained expression in the nuclei of internal and on-slide controls while pMMR was defined as retained nuclear expression in neoplastic nuclei comparable to controls. Indeterminate (iMMR) was defined as tumor nuclei showing either focal (<10% of the surface) or weak (fainter than control nuclei) expression.

**Fig. 1:**
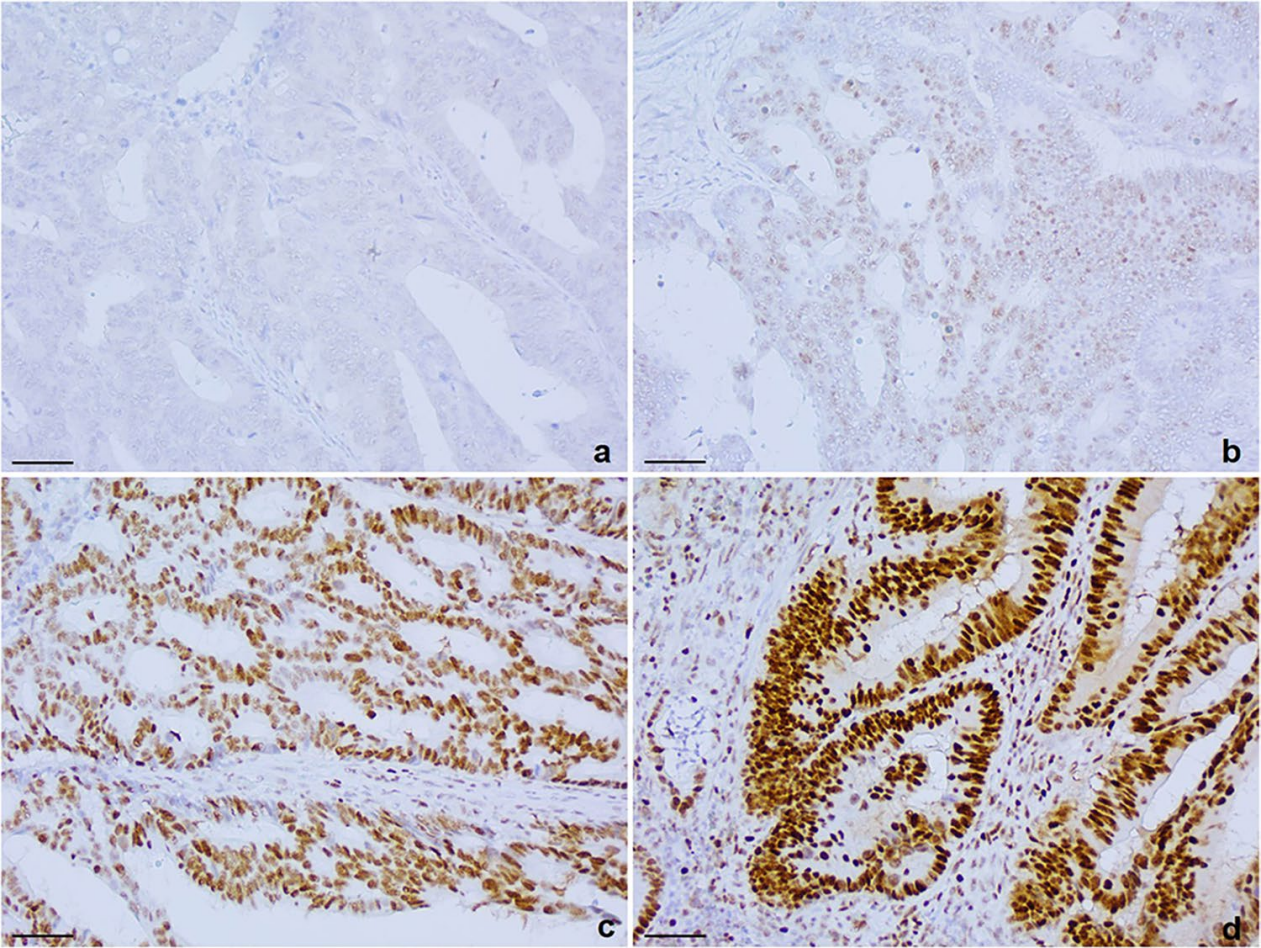
Scoring system for intensity of immunoreaction using anti-MLH1 antibody on colorectal carcinoma samples: **a**) Score 0 – absence of immunoexpression in neoplastic and internal control nuclei (scale bar 50 micron); **b**) Score 1 – faint intensity of immunoexpression in neoplastic and internal control nuclei (scale bar 50 micron); **c**) Score 2 – moderate, clearly visible immunoexpression in neoplastic nuclei, slightly more intense than internal control nuclei (scale bar 50 micron); **d**) Score 3 – intense, clearly visible immunoexpression in neoplastic nuclei, more intense than internal control nuclei (scale bar 50 micron)

IHC evaluation was performed based on the following criteria as specified in Table 2:

1. *Intensity of nuclear expression* was indicated as scores from 0 (absence of nuclear expression) to score 3 (intense nuclear immunostaining) as shown in Fig. 1 and was evaluated in control nuclei and in tumor nuclei (neoplastic nuclei were not assessed in cases of deficient MMR protein);
2. *Patchiness of staining* was expressed according to percentage areas of immunostaining. True heterogeneity, defined as areas of loss of immunoexpression in multiple adjacent glands, with preserved internal control and stark contrast between areas of preserved and deficient zones, was noted but was not evaluated as part of patchiness [22]).
3. *Presence or absence of central artefact:* this was defined as the presence of a rim of adequately stained tissue towards the outer surface but reduced/inadequate expression in the central part of the tissue [33] and was scored as absent, mild, moderate and marked.

**Table 2:** Evaluation criteria and scoring systems for MisMarch repair immunohistochemistry

MMR IHC evalutation criteria*	Score 0	Score 1	Score 2	Score 3
INTENSITY	stain not assessable; inadequate	Very Faint immunostaining	Moderate immunostaining	Intense immunostaining
PATCHINESS	Absent (staining in 100% of the sample)	Mild (staining in 70-99% of the sample)	Moderate (staining in 50-69% of the sample)	Severe (staining in 10-49% of the sample)
CENTRAL ARTEFACT	Absent	Mild (most of the section stained except for the innermost portion)	Moderate (half of the outermost portion of the section adequately stained while the innermost half was not	Marked (stain only in the outermost rim, while most of the section was weakly/inadequately stained

MMR – MisMatch Repair; IHC – immunohistochemistry

*evaluation was based on nuclear expression of the internal controls (normal tissue, lymphocytes, and stroma cells)

### Statistical analysis

Descriptive statistics was applied to demographic and histologic characteristics. The intensity, patchiness and central artefact scoring were compared between the fixation protocols and standard; differences in staining criteria between protocols for each antibody were calculated using the chi-squared test. A cut-off of *p* < 0.05 indicated a significant difference between groups.

Clinical data, including patient’s age, gender and site of tumor were obtained from the pathology database and all data were anonymized (all patients who undergo surgery in our institution sign informed consent for research purposes). Ethics committee approval was obtained at the University of Genova/IRCCS Ospedale Policlinico San Martino, Genova, Italy, number 101/2021 (1 March 2021). The study was conducted in accordance with the ethical principles of the Declaration of Helsinki.

## Results

Thirty CRC samples were collected prospectively; the case series included 9 females and 21 males (median age - 71 years; range 26–90 years). Fifteen CRCs were right sided; 9 were left sided and 6 cases were rectal cancers. With regards to stage, 12 patients were stage II, 14 were stage III, and 4 were stage IV.

The mean fixation (SD standard deviation) times were: standard fixation group (A) 31.1 hours (SD 8.99); hypo-fixation group (B) 17.27 h (SD 1.74); hyper-fixation group (C) 115,73 (SD 20.67); cold fixation (D) 26.9 h (SD 3.144).

Six-hundred immunohistochemical slides were evaluated, 150 for each antigen (MLH1, PMS2, MSH2, MSH6). Three CRCs were dMMR (loss of MLH1/PMS2 expression); the rest (27) were pMMR.

Considering all the immunoreactions performed, 46 immunoreactions (21 for MLH1, 16 for PMS2; 4 for MSH2 and 5 for MSH6) were *inadequate (score 0 intensity)* and would have required additional steps for MMR evaluation (IHC repetition or confirmatory PCR). Of the inadequate reactions, the majority were seen in the hypo-fixation group (B) – 22/59 cases (47.8%), followed by the hyper-fixation group (C) – 13/59 cases (28.1%) while cold formalin fixation (group D) showed the least inadequate cases (2/59 – 4.3%) see Table 3.

**Table 3:** Number of cases with score 0 intensity, score 2-3 patchiness and score 2-3 central artefact according to antibody (MLH1, PMS2, MSH2, MSH6) and fixation group (A-E)

Group (n° samples)	Length of fixation	Temperature	Size	MLH1 n° samples	PMS2 n° samples	MSH2 n° samples	MSH6 n° samples	Total n° samples (%)
SCORE 0 INTENSITY								
Group A (30)	Standard (24-48 hrs)	Room Temperature	Standard	3	2	0	1	6 (13.1%)
Group B (30)	Hypo (<20 hrs)	Room Temperature	Standard	12	8	1	1	22 (47.8%)
Group C (30)	Hyper (>90 hrs)	Room Temperature	Standard	3	5	2	3	13 (28.3%)
Group D (30)	Standard (24-48 hrs)	Cold (°4C)	Standard	1	0	1	0	2 (4.3%)
Group E (30)	Standard (24-48 hrs)	Room Temperature	Small	2	1	0	0	3 (6.5%)
Total				21 (45.7%)	16 (34.8%)	4 (8.7%)	5 (10.8%)	46 (100%)
SCORE 2-3 PATCHINESS								
Group A (30)	Standard (24-48 hrs)	Room Temperature	Standard	24	18	10	11	63 (24.8%)
Group B (30)	Hypo (<20 hrs)	Room Temperature	Standard	23	21	15	15	74 (29.1%)
Group C (30)	Hyper (>90 hrs)	Room Temperature	Standard	13	17	9	7	46 (18.1%)
Group D (30)	Standard (24-48 hrs)	Cold (°4C)	Standard	14	13	8	3	38 (15.0%)
Group E (30)	Standard (24-48 hrs)	Room Temperature	Small	10	11	7	5	33 (13.0%)
Total				84 (33.1%)	80 (31.5%)	49 (19.3%)	41 (16.1%)	254 (100%)
SCORE 2-3 CENTRAL ARTEFCAT								
Group A (30)	Standard (24-48 hrs)	Room Temperature	Standard	13	11	6	9	39 (25.9%)
Group B (30)	Hypo (<20 hrs)	Room Temperature	Standard	18	16	8	6	48 (31.8%)
Group C (30)	Hyper (>90 hrs)	Room Temperature	Standard	7	6	7	8	28 (18.5%)
Group D (30)	Standard (24-48 hrs)	Cold (°4C)	Standard	11	3	3	1	18 (11.9%)
Group E (30)	Standard (24-48 hrs)	Room Temperature	Small	3	6	4	5	18 (11.9%)
Total				52 (34.4%)	42 (27.8%)	28 (18.6%)	29 (19.2%)	151 (100%)

Score 2–3 *patchiness* was seen more frequently in group B (hypo-fixed cases – 74/281 - 26.3%) and group A (standard – 63/281 – 22.4%) compared to the other groups. No true case of heterogeneity was seen. *Central artefact* (score 2-3) showed similar results (group B - hypo-fixed cases – 48/157 – 30.6%) and group A - standard – 39/157 – 24.9%) as shown in Table 3.

### Assessment of single antibodies by fixation group

MLH1 performed worse compared to the other antibodies (closely followed by PMS2) especially with regard to intensity (score 0–1 versus score 2–3) of immunostaining (*p*=0.0002) in the standard fixation (group A) and even more so in the hypo-fixation group (group B) (*p*< 0.00001). MSH2 and MSH6 suffered least from problems in intensity with most cases (ranging between 86.7 and 96.7%) showing score 2–3 intensity. Patchiness and central artefact also affected MLH1 and PMS2 more than MSH2 and MSH6 (see Table 3).

### Comparison of fixation groups against standard (group A)

**Fig. 2:**
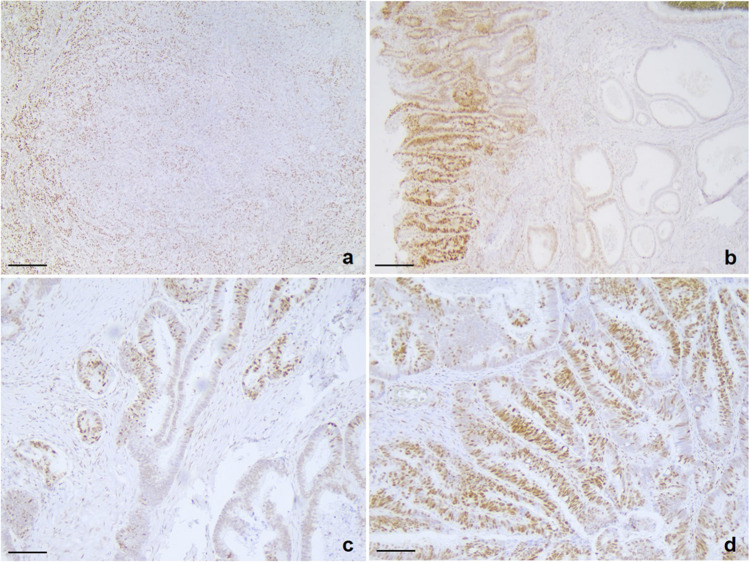
Examples of central artefact and patchiness in colorectal samples immunostained with anti-MLH1: **a**) Score 2 central artefact in a case of colorectal carcinoma in the standard group (group A) showing central area of reduced immunoexpression in a clearly proficient MisMatch Repair case (scale bar 200 micron); **b**) Score 3 central artefact in a case of colorectal carcinoma in the hypo-fixed group (group B) showing clear central area of reduced immunoexpression in a proficient MisMatch Repair case (scale bar 200 micron); **c**) Score 2 patchiness in a case of colorectal carcinoma in the hypo-fixed group (group B) in a proficient MisMatch Repair case (scale bar 100 micron); **d**) Score 1 patchiness in a case of colorectal carcinoma in the standard group (group A) in a proficient MisMatch Repair case (scale bar 100 micron)

Considering the totality of immunoreactions (irrespective of antibody) compared against the standard (group A) the following differences were observed:

- Hypo-fixation (group B) showed significantly worse immunoreaction intensity (*p*=0.0013) with increase of score 2-3 patchiness and central artefact (Fig. 2), though this was not statistically significant.
- Hyper-fixation (group C) significantly worsened the intensity of immunoexpression (*p*<0.00001) but reduced the presence of patchiness and central artefact (*p*=0.046 and *p*=0.0045 respectively) when this was considered as present (any score above 0) or absent.
- Cold fixation (group D) presented a significantly better intensity of nuclear expression (*p*=0.02) when considering score 0–1 versus score 2–3 but not when considering score 0 versus scores 1-2-3 combined; it did show reduction of patchiness (*p*=0.02), and reduced, but not significant, presence of central artefact.

### Comparison of immunoreactions between standard small block (group E) and standard (group A)

Using a small sample size did not present a significant increase in immunoreaction intensity, compared to standard size, however patchiness and central artefact were significantly reduced (*p*=0.0008 and *p*=0.0014 respectively).

## Discussion

Recent years have placed much emphasis on the impact of analytic variables on tissue biomarker interpretation using IHC. Indeed, precision medicine requires high inter-laboratory concordance for biomarker testing and the standardization of all IHC phases (pre-analytical, analytical and post-analytical) is fundamental, hence the publication of recommendations for IHC testing, validation and standardization [2, 34–36].

With regard to MMR testing, the literature is in short supply of studies on pre-analytical variables, even though they are known to be of importance [16]. One recent study showed that various tissue processing protocols did not seem to affect MMR IHC [37]; however, no detailed study on the impact of fixation on MMR testing is available. The present study is, therefore, the first to apply different fixation protocols on a prospectively collected series of CRC samples with the aim of providing a basis for future recommendations on MMR testing by IHC. The study used only fresh left-over tissue with short, annotated, cold ischemia times so as not to add a further pre-analytic variable which likely has an influence on MMR testing. Furthermore, fixation times were strictly controlled and any cases which, for any reason, did not fit into the pre-established time slots were excluded (e.g., CRC cases arriving on Friday afternoon with processing possible on Sunday night).

The most problematic antibodies (and this is well known from personal experience, though not often referred to in the literature) on the Ventana Benchmark platform are MLH1 and PMS2. This is true both for intensity of the immunosignal and patchiness of expression and this is rendered even more evident by hypo-fixation.

Indeed, hypo-fixation (<20 h for surgical specimens) and hyper-fixation (>90 h for surgical specimens) were associated with more cases of inadequate, score 0, immunoreactivity compared to the standard fixation group. Formalin creates crosslinks with proteins by forming methylene bridges between amino groups thus maintaining the tissue’s structural cohesion and inactivating lytic enzymes as well as interacting with nucleic acids. When tissues are placed in formalin, the resulting equilibrium between reactive formaldehyde species (which fix) and its non-reactive hydrate, methylene glycol (which penetrates) [38] can explain why formalin shows brisk penetration rates but slow fixation. The need for more streamlined laboratory workflow, reduced turn-around times and faster diagnosis/biomarker availability has led to the development of shorter fixation protocols, however, as shown by our study (and others in different tissue types [39]), they negatively impact IHC quality. Previous studies recommended a minimum of 8 hours of formalin [36] even though complete tissue fixation requires 24 h; this 8-h rule however, does not consider sample size and thickness as well as intra-laboratory variables. In our study, hypo-fixation, even at an average of 17 h (well above the recommended 8 hours, and even with this length of time gross samples were still slightly pink, indicating insufficient fixation), seems to cause major problems with regard to immunosignal intensity but also patchiness/central artefact of MMR expression [40].

Hyper-fixation, on the other hand can be due to various organization problems such as hub laboratories receiving partially fixed, unopened samples from different hospitals, no laboratory activity on weekends/holidays, problems in personnel leading to long turnaround times and lengthy formalin immersion, but this still shows an impact on IHC due to increased formalin cross-linking making antigens less available for immunoreactions [11, 12]. Antigen retrieval (whether temperature or enzymatic) can overcome this somewhat; however, it does mean that individual laboratories need to tailor their IHC to their own pre-analytical variables. In our study, hyper-fixation reduces central artefacts, probably due to the fact that longer immersion in formalin guarantees fixation of even the innermost tissue portions.

Cold formalin fixation (4°C) has been proposed as a valid alternative option [12, 28, 41] with superior IHC staining quality. The present study was concordant with this finding, showing improved immunosignal intensity and reduced patchiness/central artefact in samples immersed in cold formalin as it probably reduces lytic enzyme activity (preserving tissue antigenicity) and, with regard to formalin fixation, it increases its diffusion capacity in tissues. Furthermore, cold formalin fixation has been shown to better preserve DNA and RNA integrity and reduce fragmentation [28, 29]. Cold formalin fixation is probably not feasible for whole resection samples as it requires large spaces for cold storage (not available in most laboratories), however, a dedicated sample of neoplastic tissue in a cold stored biocassette could be a possibility. This would facilitate IHC evaluation and, considering the expansion of molecular biomarkers in cancer, provide high-quality DNA and RNA for PCR and NGS; however, it could unfortunately introduce further variability (especially considering that the choice of cold storage sample is made only on the basis of gross appearance).

Considering that small biopsy samples suffer from reduced impact of pre-analytic variables we decided to try and see if a small CRC sample, specifically sampled for MMR evaluation, could prove useful. The main reasoning was that the smaller the sample, the more easily formalin would penetrate and fix the tissue. Small sample size did not impact substantially on immunosignal intensity, which was similar to the standard group, however immunostaining was less patchy and more complete with little central artefact demonstrating that small sample size does permit more homogenous fixation. A future expansion on the present study would be to address problems in possible hypo-fixation of biopsy samples as they are often processed quickly to reduce turn-around times.

The main disadvantage of this study is that other pre-analytic variables were not studied, and indeed more studies analysing a wide range of pre-analytic (in particular cold ischemia, processing, storage etc [42]) and analytic factors are necessary, especially considering how important MMR testing has become for patient management/treatment.

In conclusion, this is the first study specifically created to evaluate the impact of fixation on MMR protein evaluation by IHC, showing that both hypo- and hyper-fixation with formalin can cause problems in immunosignal intensity, and therefore evaluation, and that hypo-fixation further increases patchiness and central artefacts. In short, 24-h formalin fixation is recommended (significantly longer than the previously suggested 8 h), as well as cold (4°C) formalin fixation which has shown to be a valid option for successful IHC MMR evaluation.

## Data Availability

All data are available from the corresponding author upon reasonable request.
